# Reconstruction of Sparse-View X-ray Computed Tomography Based on Adaptive Total Variation Minimization

**DOI:** 10.3390/mi14122245

**Published:** 2023-12-15

**Authors:** Zhengshan Yu, Xingya Wen, Yan Yang

**Affiliations:** College of Mechanical Engineering, Chongqing University of Technology, Chongqing 400054, China; yuzhengshan@outlook.com (Z.Y.); xingya123452021@163.com (X.W.)

**Keywords:** computed tomography (CT), sparse view, total variation, regularization parameter

## Abstract

Sparse-view reconstruction has garnered significant interest in X-ray computed tomography (CT) imaging owing to its ability to lower radiation doses and enhance detection efficiency. Among current methods for sparse-view CT reconstruction, an algorithm utilizing iterative reconstruction based on full variational regularization demonstrates good performance. The optimized direction and number of computations for the gradient operator of the regularization term play a crucial role in determining not only the reconstructed image quality but also the convergence speed of the iteration process. The conventional TV approach solely accounts for the vertical and horizontal directions of the two-dimensional plane in the gradient direction. When projection data decrease, the edges of the reconstructed image become blurred. Exploring too many gradient directions for TV terms often comes at the expense of more computational costs. To enhance the balance of computational cost and reconstruction quality, this study suggests a novel TV computation model that is founded on a four-direction gradient operator. In addition, selecting appropriate iteration parameters significantly impacts the quality of the reconstructed image. We propose a nonparametric control method utilizing the improved TV approach as a solution to the tedious manual parameter optimization issue. The relaxation parameters of projection onto convex sets (POCS) are determined according to the scanning number and numerical proportion of the projection data; according to the image error before and after iterations, the gradient descent step of the TV item is adaptively adjusted. Compared with several representative iterative reconstruction algorithms, the experimental results show that the algorithm can effectively preserve edges and suppress noise in sparse-view CT reconstruction.

## 1. Introduction

X-ray computed tomography (CT) is a non-invasive imaging technique that utilizes the attenuation data of X-rays passing through an object to reconstruct its tomographic image information. To achieve precise reconstructed images in CT systems, the projection data generally need to satisfy the Tuy–Smith data completeness condition [1,2], requiring uniform and dense acquisition over 360° or 180°. In numerous practical applications, only sparse-view sampled projection data can be used for CT image reconstruction due to constraints on data acquisition time, radiation dose, or geometric position of the imaging system scan. Sparse-view sampling refers to the acquisition of projection data with fewer angles at equal intervals within the conventional scanning range. Usually, sparse-view sampling is important in many scenarios. For example, it reduces the ionizing radiation that is harmful to human health in medical CT [3], and it enhances detection efficiency in industrial CT [4]. However, insufficient viewpoints of projection data can cause a severely ill-posed problem in image reconstruction. Even slight measurement errors can result in significant reconstruction errors, degrading the quality of the reconstructed images [5]. Therefore, research on how to obtain clinically acceptable CT images from limited projection data has become a central focus in CT image reconstruction.

For the reconstruction of images from sparse-view projection data, conventional analytical algorithms like the filtering back projection (FBP) algorithm can result in the presence of geometric distortion and streak artifacts in the reconstructed images. By contrast, iterative reconstruction algorithms, particularly when used in conjunction with regularization theory, demonstrate superior reconstruction quality in sparse-view CT compared to analytical reconstruction methods [6,7]. Sparse-view CT image reconstruction in mathematics presents an ill-posed problem. For this problem, regularization theory suggests adding a priori information as constraints to achieve a stable approximate solution. Total variation (TV) regularization [8,9], wavelet framework-based regularization [10,11], and dictionary learning [12,13] are among the prevalent methods for regularization. Choosing suitable regularization is critical to iterative reconstruction. Wavelet techniques excel in preserving edge and low-contrast data, while TV methods are efficient in reducing noise and streak artifacts. Wavelet-based iterative approaches often integrate TV regularization, or deep learning, which demands additional storage capacity and computational time [14,15,16]. Sparse-view CT image reconstruction using dictionary learning typically involves acquiring a training sample set either from CT images reconstructed using the FBP algorithm in a standard dosage scenario, or by segmenting intermediate reconstructed images during each iteration. This method is computationally intensive and time-consuming [13]. Instead of consuming expensive computational costs, we note that traditional TV regularization methods can also produce good results in the reconstruction of sparse view images by combining the sparsity of image gradient amplitude. However, the conventional TV algorithm features a singular or fixed direction in its gradient operator, leading to the potential loss of edge and detail information in the reconstructed image for sparse-view CT. This paper aims to focus on the TV algorithm for CT image reconstruction in sparse view and achieve finer image restoration utilizing fewer projection data.

Earlier, Candes et al. [17,18] proposed the theory of compressed sensing combined with the TV minimization method to achieve high-quality CT image reconstruction with limited projection data. In 2006, Sidky et al. [8] proposed a reconstruction algorithm based on projection onto convex sets with TV minimization (POCS-TV), which uses the POCS algorithm as a data fidelity term, adds a TV term to constrain image convergence during the reconstruction process, and solves the optimization problem by using the steepest-descent method to achieve CT image reconstruction from sparse-view projection data. In 2008, Sidky et al. [9] improved the POCS-TV algorithm and proposed the adaptive-steepest-descent (ASD-POCS) algorithm. In this method, the TV objective is minimized via the adaptive-steepest-descent procedure. While the traditional TV technique reduces noise and artifacts effectively, due to the isotropy of the TV regularization term and the single direction of the gradient, it is easy to make the edges and details of the image too smooth [19]. Researchers have proposed various TV methods to enhance reconstructed image quality, such as the edge-preserving total variation (EPTV) algorithm [20], the adaptive-weighted TV (AwTV) algorithm [21], and the generalized anisotropic total variation minimization method [22]. However, these algorithms solely take into account the vertical and horizontal directions for the gradient operator of the TV term and ignore the diagonal direction. To address this limitation, Deng et al. [23] proposed a diagonal TV computation method in 2015 that only considers the diagonal gradient in the model, claiming to reconstruct a better image than the traditional TV method that only considers the vertical and horizontal directions. Additionally, researchers have proposed TV models based on different directions and multi-directions to better recover edges and details lost in minimized TV, including TV models based on 8- and 26-directional gradient operators [24], multi-direction anisotropic total variation (MDATV) [25], and a BDTV model that adaptively computes the orientation information of gradient operators [19]. Compared to other reconstruction algorithms, the TV models utilizing multi-directional gradient operators are the most time-consuming. To prevent edge degradation due to the single-directional TV regularization gradient and computational speed loss from an excess of directional gradient operators, we suggest exploring a research method that includes four directional gradient operators—vertical, horizontal, and diagonal directions.

Mahmoudi et al. [26] combined adaptive-weighted total variation with adaptive-weighted diagonal total variation (AwTV + AwDTV) in 2019. In this method, AwTV considered vertical and horizontal gradients, while AwDTV considered diagonal gradients. AwTV and AwDTV are multiplied by the weight and then added to form a TV regularization term. So how to select the weight becomes key to the algorithm. It often needs a time-consuming trial-and-error process to determine the optimal weight. In this study, we propose a new four-direction computed TV term gradient operator to retain more image edge information.

Most iterative algorithms typically require numerous experiments to select empirical parameters to maintain a balance between the data fidelity term and the TV regularization term. Failure to do so may affect the convergence speed of the iteration and the quality of the reconstructed image. To avoid manual selection, researchers have utilized ant colony optimization algorithms [27] and machine learning [28] in conjunction with CT image quality assessment metrics to select appropriate parameters. All of these require a huge amount of computation to obtain satisfactory-quality reconstructed images.

To avoid tedious manual parameter tuning, we propose a nonparametric control method based on the improved TV method, which determines the relaxation parameter in POCS based on the noise-level characteristics of the projection data, and determines the step size parameter in TV minimization based on the projection error adaptation of the reconstructed image during POCS iteration.

The paper is structured as follows: Section 2 presents the CT image reconstruction model for sparse view, followed by a description of the developed reconstruction algorithm. Section 3 provides an overview of the experimental results. A discussion is provided in Section 4, followed by conclusions in Section 5.

## 2. Methods

### 2.1. CT Imaging Model with Conventional TV Minimization

Theoretically, both normal-view and sparse-view CT imaging models can be approximated as the following discrete linear system [29]:

(1) P=WF,

where $F={\left[f_{1},f_{2},\ldots ,f_{N}\right]}^{T}$ represents the image vector, $P={\left[p_{1},p_{2},\ldots ,p_{M}\right]}^{T}$ denotes the measurement vector that presents the X-ray projection, and $W=\{w_{i,j}\}$ represents the projection matrix of size *M* × *N*. *M* and *N* denote the total number of projections and image pixels, respectively. In this study, the weight factor $w_{i,j}$ of the projection matrix *W* is calculated using the grid traversal method [30] based on the intersection length of the *j*-th ray passing through the *i*-th pixel. For sparse-view CT imaging, the number of measured projection data is significantly smaller than the number of image pixels. Thus, Equation (1) belongs to an ill-posed problem, and it is difficult to obtain accurate CT images. To obtain a satisfactory approximate solution, various regularization-based optimization models have been proposed. The TV regularization-based reconstruction algorithm model can be demonstrated as:

(2) $$F^{*}=\arg\min\frac{1}{2}||WF-P|{|}_{2}^{2}+\eta ||F|{|}_{TV},$$

where the first term is the data fidelity term, the second term is the TV regularization term containing a priori information about the reconstructed image, and *η* is a parameter balancing the data fidelity term and the TV regularization term. By adding TV minimization with non-negative image values as the basic constraint, the following constrained optimization problem will be obtained:

(3) $$F^{*}=\arg\min||F|{|}_{TV},\;\mathrm{subject}\;\mathrm{to}\;P=WF,\;F\geq 0.$$

**Figure 1:**
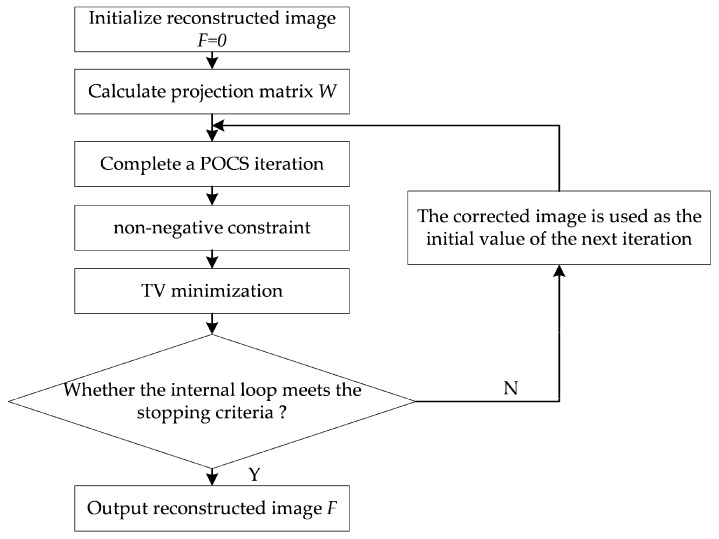
Flowchart for POCS-TV reconstruction.

The optimal solution is obtained for the above constrained optimization problem using two-stage alternating iterations. As shown in the flowchart in Figure 1, in the first stage, POCS is used: the data fidelity term projects the estimated image forward to obtain the computed projection, and then the difference between the computed projection and the actual projection is projected back into the image space to update the estimated image [8]. The second stage employs the gradient descent method to minimize TV. These two phases are alternately solved through several iterations until the iteration condition is met.

### 2.2. TV Model of Multidirectional Gradient Operators

**Figure 2:**
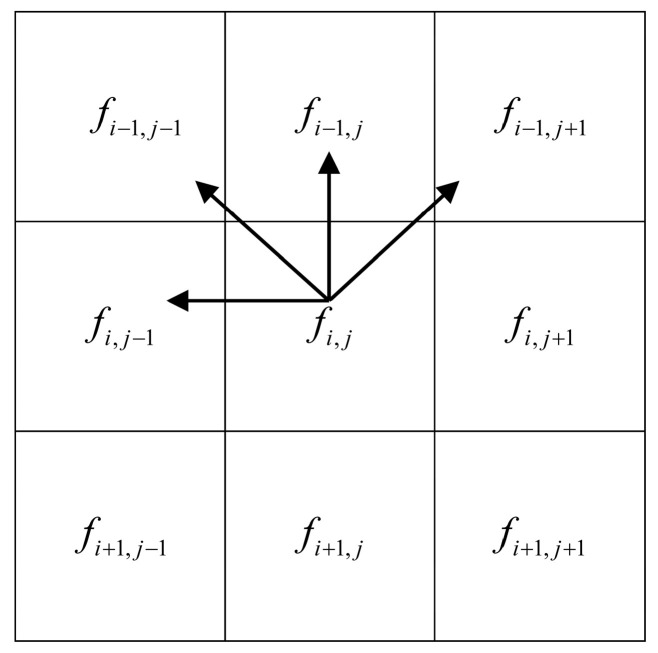
An illustration of pixel position in an image.

A pixel in image *F* is denoted by $f_{i,j}$, where *i* and *j* refer to the row and column of the image matrix, as shown in Figure 2.

A conventional TV model calculates the gradient operator only in the horizontal and vertical directions, leading to the loss of edges and details when minimizing TV [31]. To better preserve the edges and details of the projection data in the missing directions, we introduce the gradient operator in the diagonal direction and propose a four-directional gradient operator for the TV mode. As shown by the arrow in Figure 2, it represents the horizontal, vertical, and diagonal directions of the gradient operator in the two-dimensional plane. Although Yu et al. [32] proposed a four-directional gradient operator calculation method, the method considered the horizontal left and right directions and the vertical up and down directions. We used Yu et al.’s mathematical derivation method to obtain the following approximation for our model’s gradient operator:

(4) $$\mu_{i,j}=\sum_{i,j}\sqrt{\frac{{\left(f_{i,j}-f_{i-1,j}\right)}^{2}+{\left(f_{i,j}-f_{i,j-1}\right)}^{2}+{\left(f_{i,j}-f_{i-1,j-1}\right)}^{2}+{\left(f_{i,j}-f_{i-1,j+1}\right)}^{2}}{2\Delta^{2}}+\varepsilon^{2}},$$

where Δ is the sampling interval and *ε* is a constant added to the calculation of the steepest-descent direction to prevent the denominator from being zero. In this paper, we take the value of *ε* to be 10^−8^. Thus, the TV expression of the CT image is ${\left||F|\right|}_{TV}=\sum_{i,j}\mu_{i,j}$, and its gradient is calculated as follows:

(5) $$\frac{\partial ||F_{TV}||}{\partial f_{i,j}}=\frac{4f_{i,j}-f_{i+1,j}-f_{i-1,j}-f_{i,j+1}-f_{i,j-1}}{u_{i,j}}+\frac{f_{i,j}-f_{i+1,j}}{u_{i+1,j}}+\frac{f_{i,j}-f_{i-1,j}}{u_{i-1,j}}+\frac{f_{i,j}-f_{i,j+1}}{u_{i,j+1}}+\frac{f_{i,j}-f_{i,j-1}}{u_{i,j-1}}.$$

### 2.3. The Process of POCS Reconstruction

In the POCS stage, this paper applies the simultaneous algebraic reconstruction technique (SART) to achieve plausible resolution for the data fidelity term. The SART algorithm is an advancement of the algebraic reconstruction technique (ART) algorithm, which utilizes the errors of all rays that pass through a pixel at the same projection angle to determine the correction value to be applied to that pixel, rather than considering only one ray. This is equivalent to smoothing the noise in ART and improves the reconstruction efficiency [33]. The iterative formula for SART is

(6) $$f_{j}^{\left(k\right)}=f_{j}^{\left(k-1\right)}+\lambda \frac{\sum_{p_{i}\in P_{\phi }}\frac{p_{i}-\sum_{n=1}^{N}w_{in}f_{n}^{\left(k-1\right)}}{\sum_{n=1}^{N}w_{in}}w_{ij}}{\sum_{p_{i}\in P_{\phi }}w_{ij}},$$

(7) $$F_{SART}^{\left(k\right)}={\left[f_{1}^{\left(k\right)},f_{2}^{\left(k\right)}\ldots ,f_{j}^{\left(k\right)},\ldots ,f_{N}^{\left(k\right)}\right]}^{T},$$

where *k* represents the number of iterations, *i* is the ray number (1≤i≤M), *j* is the pixel number (1≤j≤N), *p_i_* is the projection value of the *i*-th ray, $P_{\phi }$ is the set of projection values at the same projection angle, $\sum_{i}w_{ij}$ represents the sum of the columns corresponding to pixel *j*, and $\sum_{n=1}^{N}w_{in}$ represents the sum of the rows corresponding to ray *i*. λ is a relaxation parameter that usually needs to be found to its optimal value through a large number of experiments.

Liu et al. [34] approximated the noise variance of X-ray photon detection during ART by utilizing Poisson statistics-based projection values. The inverse of each ray’s projection value is expressed as the noise variance of projected image pixel points, thereby determining the relaxation parameter for each pixel point in the iterative process. The reconstruction of different pixels requires different relaxation parameters. However, noticeably patchy shadows can result from significant differences in relaxation parameters between neighboring pixel points during image reconstruction. This paper proposes an improved method to determine relaxation parameters by utilizing the ratio between the projected mean value of all rays under the same projection angle and the projected mean value of the entire projection data in the SART reconstruction process. The following formula is used for calculation:

(8) $$\lambda_{\phi }=\sqrt{\exp\left(-\sum_{p_{i}\in P_{\phi }}p_{i}/\sum_{i=1}^{M}p_{i}\right)}.$$

It can be seen that $\lambda_{\phi }$ is constrained to be at [0, 1]. The effect of the relaxation parameter of SART on the reconstruction is described in the experiments in Section 3.3. To reinforce the non-negativity constraint, any negative values within the reconstructed image are assigned to zero. The POCS stage in this paper involves incorporating both non-negativity projection and SART.

### 2.4. TV Minimization Using Adaptive Step Size

The steepest-descent method is employed to minimize the TV function. Starting from the initial point, the iterative formula for the steepest-descent method is:

(9) $$F^{\left(n\right)}=F^{\left(n-1\right)}-\eta \frac{G^{\left(n-1\right)}}{\left|G^{\left(n-1\right)}\right|},$$

where $G^{\left(n-1\right)}=(\partial ||F^{\left(n-1\right)}|{|}_{TV}/\partial f_{i,j})$ and *n* represents the number of iterations for the TV minimization process. The gradient descent step size, η, is a crucial parameter for balancing the POCS process and TV minimization.

In the ASD method proposed in the literature [9], the gradient descent step is adapted based on the alterations in the image during the POCS stage. However, the method necessitates that users establish multiple parameters to govern gradient descent step-size control. This empirical process limits the method’s appeal. Building on this approach, our paper enhances the adaptive scheme through image domain transformations and posits a nonparametric control scheme:

(10) $$\eta =\frac{\Delta_{d}^{\left(1\right)}}{\sum_{k=1}\Delta_{d}^{\left(k\right)}}\times \Delta_{d}^{\left(k\right)}$$

where $\Delta_{d}^{\left(k\right)}={\left||F_{SART}^{\left(k\right)}-F^{\left(k-1\right)}|\right|}_{2}$ represents the image change initiated by the POCS stage, $F_{SART}^{\left(k\right)}$ denotes the reconstructed image after the *k*th iteration of the POCS stage, $F^{\left(k-1\right)}$ represents the reconstructed image following the *k*-1th iteration of the POCS-TV main loop, and $\Delta_{d}^{\left(1\right)}$ represents the image change after the first iteration of the POCS stage. The optimization process results in the reconstructed image *F* converging to a feasible solution. As the number of POCS-TV iterations increases, it is necessary to update the smaller step size [9]. In this approach, the step size is adjusted completely by the change in the image updated by POCS at each iteration. The step size begins with $\Delta_{d}^{\left(1\right)}$ and gradually decreases as the iteration progresses. Following multiple iterations, the reconstructed image approaches the feasible solution. Compared to ASD-POCS, our method eliminates the need for artificial parameters and removes the burden of empirical parameter selection.

### 2.5. Stopping Criterion

To ensure that the above algorithm’s solution for the objective function (3) is an optimal estimate, this paper uses the stopping criterion proposed in the literature [35]. This criterion uses the difference between images from consecutive iterations as an indicator of the algorithm’s convergence.

(11) $$res=\frac{1}{N}{\left||F^{\left(k\right)}-F^{\left(k-1\right)}|\right|}_{2},$$

where *N* is the total number of image pixels. As the number of iterations increases, *res* converges to zero. This paper adopts *res* < 10^−12^ as the iteration stop criterion.

In summary, the pseudo-code of the algorithm proposed in this paper is as follows (Algorithm 1):
**Algorithm 1. Implementation Steps of Our Algorithm**Input: $F^{0}=0,\;K_{iter},\;N_{TV}=20,\;\varepsilon_{0}=10^{-12}$ For $k=1,2,\ldots ,K_{iter}$ For $\phi =1,2,\ldots ,N_{view}$ SART Updating according to Formula (6)–(8) $f_{j}^{\left(k\right)}=\left\{\begin{array}{c} f_{j}^{\left(k\right)},\;f_{j}^{\left(k\right)}\geq 0\\ 0,\;f_{j}^{\left(k\right)}<0 \end{array}\right.$ End TV Minimization: $\Delta_{d}^{\left(1\right)}={\left||F^{\left(1\right)}-F^{\left(0\right)}|\right|}_{2},\;\Delta_{d}^{\left(k\right)}={\left||F_{SART}^{\left(k\right)}-F^{\left(k-1\right)}|\right|}_{2},\;\eta =\frac{\Delta_{d}^{\left(1\right)}}{\sum_{k=1}\Delta_{d}^{\left(k\right)}}\times \Delta_{d}^{\left(k\right)},\;F_{TV}^{\left(0\right)}=F_{SART}^{\left(k\right)}$ For $n=1,2,\ldots ,N_{TV}$ $G^{\left(n-1\right)}=\frac{\partial ||F_{TV}^{\left(n-1\right)}||}{\partial f_{i,j}},\;G^{\left(n-1\right)}=\frac{G^{\left(n-1\right)}}{\left|G^{\left(n-1\right)}\right|},\;F_{TV}^{\left(n\right)}=F_{TV}^{\left(n-1\right)}-\eta \frac{G^{\left(n-1\right)}}{\left|G^{\left(n-1\right)}\right|}\;$ End $F^{\left(k\right)}=F_{TV}^{\left(n\right)},\;res=\frac{1}{N}{\left||F^{\left(k\right)}-F^{\left(k-1\right)}|\right|}_{2}$ If $res<\varepsilon_{0}$, break; end End Output: reconstructed image *F*

## 3. Experimental Results and Discussion

To validate the effectiveness of our algorithm in the case of sparse-view CT imaging, we investigate the performance of the proposed method using a digital phantom and a physical phantom. We compare our proposed method to SART, SART-TV, SART-FDTV, and POCS-ASD. SART-FDTV’s TV minimization was computed using the four-direction gradient operator based on our proposed method explained in this paper. The hardware and software environments for the experiments are Matlab R2022b, Intel(R) Core(TM) i7-13700H-2.40 GHz, 32 GB RAM, and Windows 11 64-bit system. To assess the performance of different reconstruction algorithms, structural similarity (SSIM) [36] and peak signal-to-noise ratio (PSNR) are used for quantitative analysis.

(12) $$\mathrm{PSNR}=10\log_{10}\frac{{\left(\max(f_{i,j}^{*})\right)}^{2}}{\frac{1}{M\times N}\sum_{0<i<M}\sum_{0<j<N}(f_{i,j}-f_{i,j}^{*}{)}^{2}},$$

where $f_{i,j}$ and $f_{i,j}^{*}$ indicate the pixel values of the original and reconstructed images.

(13) $$SSIM=\frac{\left(2\mu_{O}\mu_{R}+C_{1})(2\sigma_{O}\sigma_{R}+C_{2}\right)}{\left(\mu_{O}^{2}+\mu_{R}^{2}+C_{1})(\sigma_{O}^{2}+\sigma_{R}^{2}+C_{2}\right)},$$

where $\mu_{O}$ and $\mu_{R}$ represent the mean values of the original and reconstructed images and $\sigma_{O}$ and $\sigma_{R}$ represent their standard deviations. Small positive constants *C*_1_ and *C*_2_ are used to prevent instability when the denominators are close to zero. The SSIM index evaluates the visual impact of an image’s three attributes: brightness, contrast, and structure. A higher SSIM value indicates a finer reconstructed image.

### 3.1. Digital Phantom

In this section, the Shepp–Logan phantom is used as a simulation model to evaluate the effectiveness of the improved algorithm in sparse-view CT reconstruction. The image size of the reconstruction is 256 × 256, and the number of X-ray detectors is 512. The amount of projection data is the crucial factor influencing the reconstruction results in sparse-view reconstruction. In this experiment, we collected data by sampling at equal intervals over an area of 180° to obtain projection data at 30, 60, and 90 projection angles. The algorithm parameters and number of iterations are chosen through empirical methods for comparison in iterative algorithms. The SART and ART reconstruction processes are assigned a relaxation parameter λ value of 0.5. The value of the gradient descent step size of the TV minimization process in the SART-TV and SART-FDTV algorithms, α, is 0.3. The total number of iterations of the algorithm is set to $K_{iter}=200$, and the number of iterations of TV minimization NTV is set to $K_{TV}=20$.

**Figure 3:**
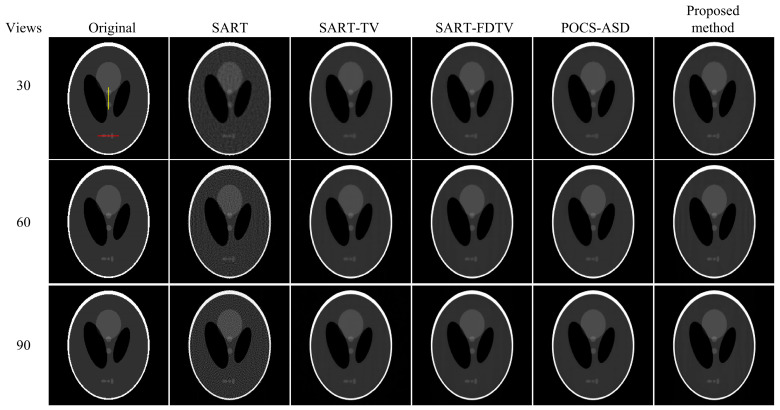
Reconstruction results of digital phantom from 30, 60, and 90 views.

**Figure 4:**
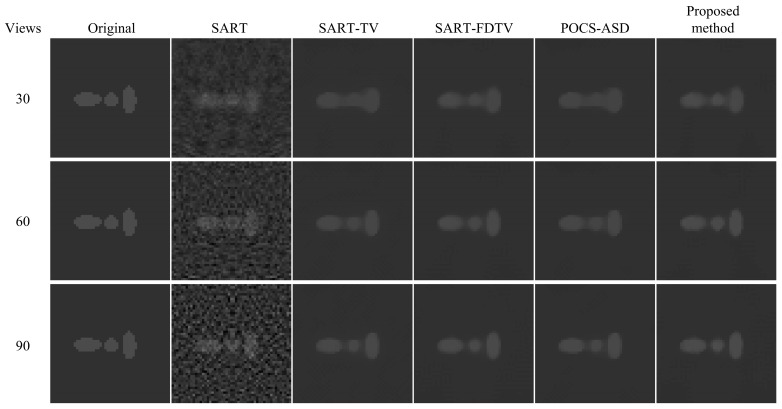
A detailed section of Figure 3.

The results of the reconstruction with different algorithms are shown in Figure 3. In Figure 4, the zoomed region of interest (ROI) corresponding to the reconstruction results in Figure 3 is shown. From Figure 3 and Figure 4, it can be observed that under sparse-view projection, the smooth regions of the reconstructed images from SART show severe noise and streak artifacts and the SART reconstructed images become blurred as the number of projection views decreases. In contrast, despite using fewer projection data, the TV method is capable of capturing most of the structure, resulting in enhanced visualization. When reducing the number of projection views to 30 and comparing the reconstruction results of SART-TV and SART-FDTV under the same empirical parameters, the SART-FDTV method exhibits a significant edge preservation improvement. The experiments show that the FDTV minimization technique proposed in this study is effective in suppressing noise and preserving more structural details during sparse-view reconstruction. Furthermore, the SART-FDTV algorithm, which is based on the non-parametric control procedure presented in this paper, also yields satisfactory reconstruction outcomes.

To further quantify the reconstruction effect, Table 1 presents the PSNR and SSIM results for various scan angle ranges. We have observed an interesting phenomenon as when the number of projection views increases, the edges of the SART reconstructed images become clearer, but there is obvious noise in the smooth area. Conversely, blurrier SART reconstructed images exhibit a better quality index when projection views are fewer. While the TV-based algorithm effectively suppresses noise and artifacts, and its quality index increases with the range of scanning angles, it can be seen that the reconstruction results of our algorithm have the best quality index. To be more precise, the SSIM results indicate that the proposed method proficiently preserves finer structural details, and the PSNR results reveal that the method in this paper is more closely aligned with those of the reference image.

**Figure 5:**
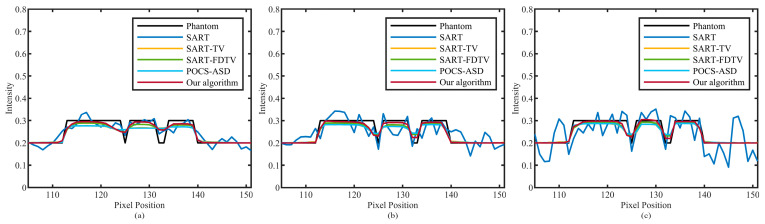
The profile of the horizontal red line in Figure 3. The numbers of sparse views of (**a**–**c**) are 30, 60, and 90.

**Figure 6:**
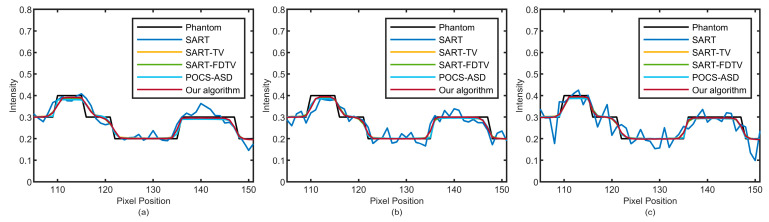
The profile of the vertical yellow line in Figure 3. The numbers of sparse views of (**a**–**c**) are 30, 60, and 90.

The improved algorithm’s performance is further confirmed by plotting profiles. As shown in Figure 3, one line lies along the horizontal direction (red horizontal line), and the other line lies along the vertical direction (yellow vertical line). The analysis results are shown in Figure 5 and Figure 6. The results of 30, 60, and 90 projection views are presented from left to right columns. It can be seen that the amplitude of the SART algorithm fluctuates greatly and deviates from the real value of pixels. All TV-based algorithms outperform the SART algorithm. Furthermore, our algorithm produces more compatible results with the original model and more accurately reconstructs corresponding images.

### 3.2. Physical Phantom

**Figure 7:**
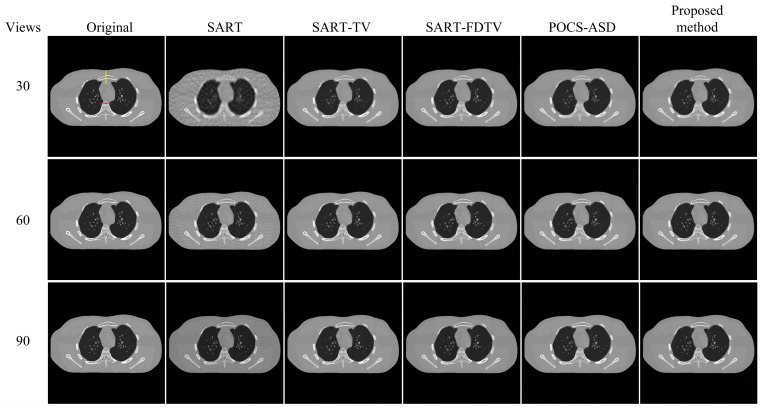
Reconstruction results of physical phantom from 30, 60, and 90 views.

**Figure 8:**
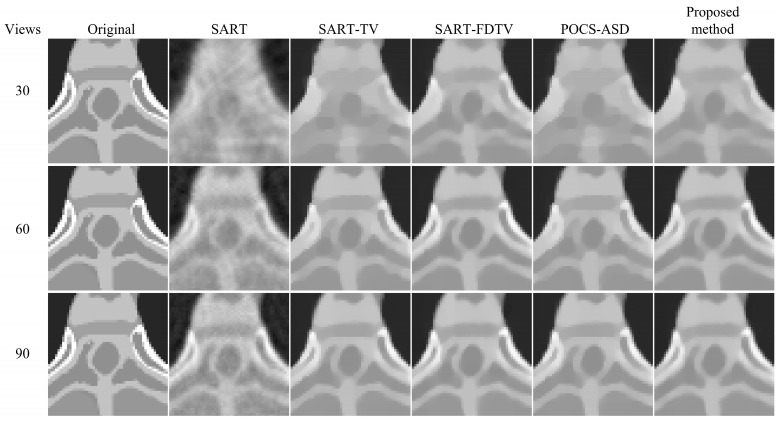
A detailed section of Figure 7.

In this section, we further assess the algorithm’s effectiveness through a medical model [37]. The reconstructed image size is 420 × 420 and pixel size is $\Delta_{x}=\Delta_{y}=1.1667\;\mathrm{mm}$. The projection view’s data acquisition process remains identical to the previous experiment, with projection data obtained at 30, 60, and 90 projection angles. The five algorithms used for contrast are the SART algorithm, the SART-TV algorithm, the SART-FDTV algorithm, the POCS-ASD algorithm, and our algorithm. The reconstruction parameter settings for all five algorithms are the same as in the previous experiment. Figure 7 and Figure 8 show the reconstruction results of the five iterative algorithms and the enlarged ROI views, respectively. Upon comparison, our algorithm exhibited superior sparse-view CT reconstruction quality.

**Figure 9:**
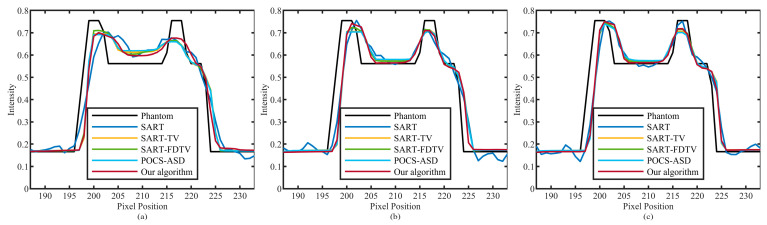
The profile of the horizontal red line in Figure 7. The numbers of sparse views of (**a**–**c**) are 30, 60, and 90.

**Figure 10:**
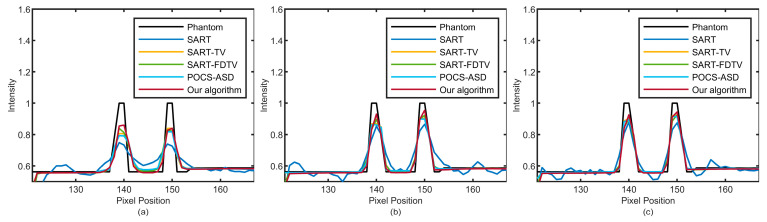
The profile of the vertical yellow line in Figure 7. The numbers of sparse views of (**a**–**c**) are 30, 60, and 90.

The PSNR and SSIM results for each of the five algorithms are presented in Table 2. Our algorithm exhibits the highest quality index while maintaining optimal performance. The profile of the horizontal line in Figure 7 is visible in Figure 9, and the profile of the vertical line in Figure 7 is visible in Figure 10. The reconstruction results of our algorithm are observed to be the most similar to actual values as shown in the profiles. The results from the physical model testing also demonstrate the advantages of our algorithm in terms of artifact reduction, edge protection, and noise suppression.

### 3.3. Effect of The Parameters

To further clarify how the relaxation parameter of POCS and the gradient descent step size of TV minimization impact the quality of reconstructed images, and to demonstrate the superiority of our proposed nonparametric control method, we vary the relaxation parameter and gradient descent step size of SART-TV and SART-FDTV in our experiments. Although the optimal gradient descent step size is suggested to be 0.2 in the literature [9], for additional comparative illustration, we set the gradient descent step size and relaxation parameter to three combinations: “1 + 0.2”, “0.1 + 0.2”, and “1 + 1”. The CT image reconstruction is performed using 30 projection views.

**Figure 11:**
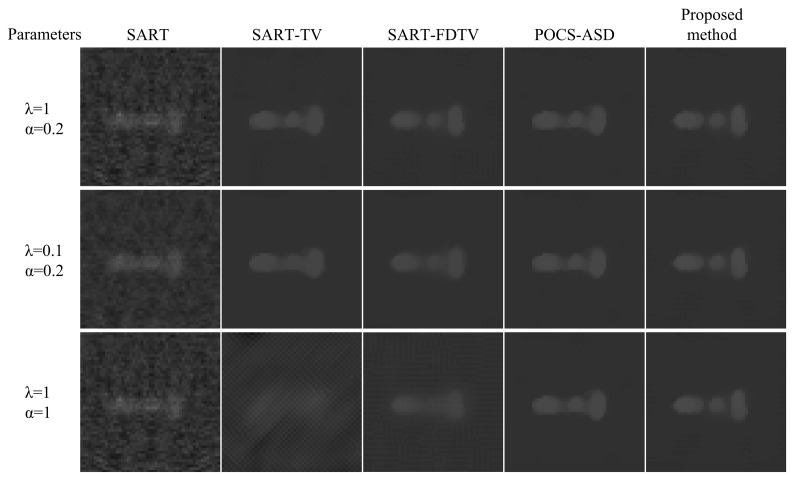
ROI of the Shepp–Logan phantom reconstructed with 30 sparse views.

**Figure 12:**
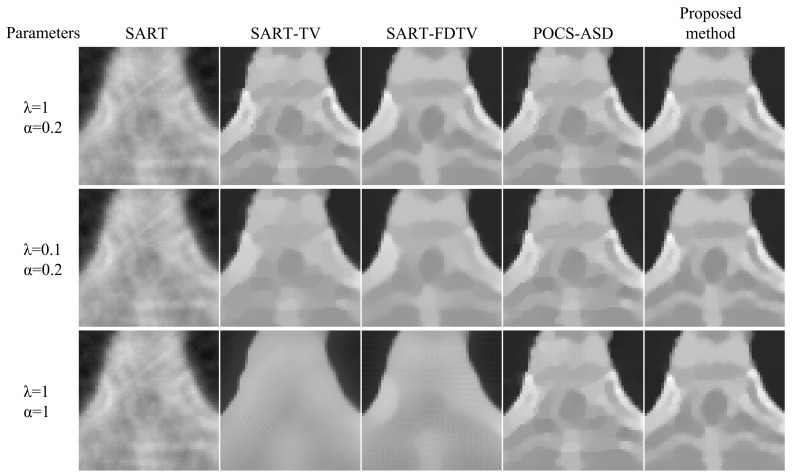
ROI of physical phantom reconstructed with 30 sparse views.

The experimental results of the enlarged region of interest (ROI) of the Shepp–Logan phantom are shown in Figure 11. Comparing the reconstructed images of the first and second rows in Figure 11, the edges of the images are slightly blurred after changing the relaxation parameter λ from 1 to 0.1 for SART-TV and SART-FDTV. Comparing the reconstructed images in the first and third rows of Figure 11, the edges of the images are severely degraded after the gradient descent step is changed from 0.2 to 1. In comparison, the nonparametric control method proposed in this paper restores a finer image. The effectiveness of our method is also verified by the medical model reconstruction image results presented in Figure 12.

**Figure 13:**
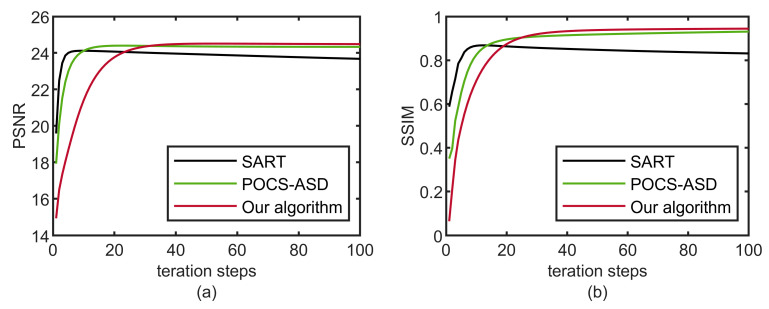
PSNR and SSIM of the Shepp–Logan phantom reconstructed with 30 sparse views. (**a**) PSNR. (**b**) SSIM.

In Figure 11 and Figure 12, the reconstructed images from the adaptive-steepest-descent (ASD-POCS) algorithm and the proposed nonparametric control method do not have a very significant visual difference. The PSNR and SSIM variations of the reconstructed images were graphed as iterative curves (see Figure 13). We can observe that the proposed algorithm maintains superior PSNR and SSIM levels, albeit converging at a slower rate than ASD-POCS.

## 4. Discussion

### 4.1. The Gradient Operator for TV Regularization

Sparse-view computed tomography (CT) refers to CT techniques whereby the number of measured projection data is significantly lower than that of traditional CT scans. Mathematically, sparse-view image reconstruction poses challenges due to its ill-posed nature. When utilizing the conventional SART method in sparse-view CT, noise and artifacts are evident in both simulation and experimental results. Sidky et al. demonstrated that incorporating TV minimization into the iterative reconstruction process can effectively eliminate high-frequency components, such as noise and artifacts, produced in sparse-view imaging. However, the experimental results indicate that as the number of projected views decreases, the edges of CT images reconstructed using the classical SART-TV algorithm become increasingly blurry. The edge structure of clinical images has a significant impact on the diagnosis and treatment of critical medical conditions [38]. It is necessary to reconstruct more detailed CT images.

The direction of the gradient operator in the classical TV method only considers the vertical and horizontal directions of the two-dimensional plane. Artifacts may appear along directions that are not tangent to projection rays, as Quinto [31] has demonstrated. Thus, artifacts are tied to direction. Various improved TV methods based on gradient direction have also demonstrated that considering more gradient directions in the iterative process can effectively enhance edges and details. Different from the calculation methods of another four-directional gradient operator [32], our proposed four-directional gradient operator also verifies this conclusion by considering the vertical, horizontal, and diagonal directions.

From the enlarged region of interest (ROI) in Figure 4 and Figure 8, it can be seen that the reconstructed images in 60 views and 90 views using the proposed four-direction gradient operator FDTV algorithm are not significantly different from other TV algorithms in terms of visual image edge. However, quantitative analysis in Table 1 and Table 2 shows that the FDTV algorithm achieves higher PSNR and SSIM values. Under the projection data of 30 views, the image reconstructed by the FDTV algorithm is visually clearer. These experimental results verify the effectiveness of our proposed four-directional gradient operator in sparse-view CT image reconstruction.

### 4.2. Iterative Parameter Analysis

**Figure 14:**
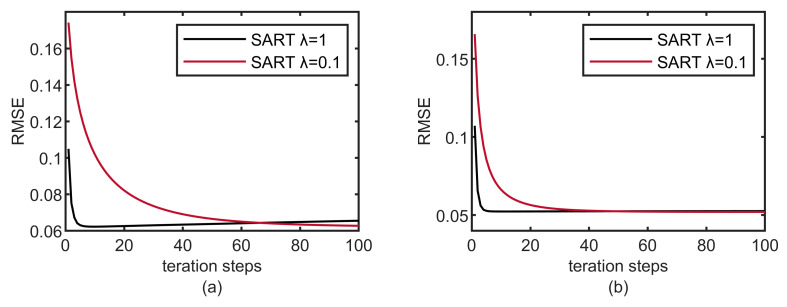
RMSE of the phantom reconstructed with 30 sparse views. (**a**) The Shepp–Logan phantom; (**b**) physical phantom.

The iterative reconstruction algorithm typically involves several parameters that significantly impact the quality of the reconstructed image. The relaxation parameters affect the convergence rate of SART. As can be seen from the root mean square error (RMSE) curve reconstructed by SART (Figure 14), the convergence rate of lower relaxation parameters is slow. However, when noise is high, using lower relaxation parameters can lead to better reconstruction (i.e., lower RMSE) [34]. The reduction of the projection view leads to an ill-posed problem, which is equivalent to introducing noise into CT reconstruction under the condition of data completeness. Therefore, in reducing the adverse effects of noise increase, lower relaxation parameters will make POCS reconstruction perform better. Obviously, in Figure 14, the SART algorithm with a relaxation parameter of 0.1 converges to a lower RMSE, which also confirms this observation. In our nonparametric control method, the relaxation parameter is limited to the range of [0, 1] by statistical projection data distribution. When the projection view is reduced, the relaxation parameter obtained by this method decreases, which is more beneficial for image reconstruction.

The gradient descent step α balances data fidelity and regularization, playing a key role in preserving edges and removing artifacts. Sidky et al. [9] pointed out that when the number of POCS-TV iterations becomes larger, the smaller step size should be updated. When the value of α remains large during the iteration process, the image will become blurred or mottled. When α is too small, noise and stripes appear in the reconstructed image.

As can be seen from Figure 11 and Figure 12, when a higher value of α is used, the reconstructed images of the SART-TV and SART-FDTV algorithms are severely blurred. When the value of α is too small and tends to 0, it is equivalent to weakening the SART algorithm of the TV term. Obviously, the experimental result shows the disadvantage of the SART algorithm in noise suppression in sparse-view CT image reconstruction. Our method adaptively adjusts the gradient descent step by utilizing the update in the image domain. With an increase in the number of iterations, the step size tends to approach 0. The above experimental results effectively verify that this method can restore a more refined image in sparse-view CT reconstruction. Although the iterative reconstruction algorithm can yield superior results, its slow reconstruction speed remains a significant limitation, which will be the focus of our future research.

## 5. Conclusions

Despite the rapid development of CT imaging technology, the reconstruction of incomplete projection data remains a significant challenge in the field. The present study developed a sparse-view CT image reconstruction algorithm based on adaptive total variation (TV) minimization. The algorithm first uses a TV regularization (FDTV) term calculated based on four directional gradients to replace the ordinary TV regularization term, and then applies it to the SART algorithm. The four gradient directions of the FDTV term take into account the vertical, horizontal, and diagonal directions of the two-dimensional plane of the image. Using simulation data and clinical data, we sampled the projection data of 30, 60, and 90 views at equal intervals in the range of 180 ° for reconstruction, and compared them with the SART and SART-TV algorithm. With the reduction of the number of projections, the experimental results verified that this method can restore finer images visually and in the quantitative evaluation of PSNR and SSIM.

Reasonable selection of iteration parameters plays a key role in the quality of the reconstructed image. In addition, we propose a nonparametric control method based on the FDTV method. In the SART iteration process of the POCS stage, the relaxation parameter of each iteration is limited to [0, 1] by calculating the ratio between the mean value of all ray projection data and the mean value of all projection data at each angle. When there are fewer projected views, the relaxation parameters obtained by our method are lower. Although the lower relaxation parameter sacrifices the convergence speed of the algorithm, it will be more conducive to image reconstruction. In the TV stage, if the gradient descent step value is kept too large or too small, the effect of the reconstructed image is not ideal. We use the image changes before and after POCS to adjust the step size. As the number of iterations increases, the error of image change before and after iteration decreases, and the step size tends to 0. When there are fewer projected views, the reconstruction results show that the proposed algorithm has better performance than POCS-ASD in structure information preservation and artifact suppression.

## Figures and Tables

**Table 1:** SSIM and PSNR of the reconstruction results of digital phantom.

Number of Views	Index	SART	SART-TV	SART-FDTV	POCS-ASD	Our Algorithm
30	PSNR	23.7308	23.9988	24.1963	24.0068	24.2794
	SSIM	0.8399	0.9077	0.9359	0.9320	0.9498
60	PSNR	22.4447	24.2463	24.4704	24.2596	24.5714
	SSIM	0.7556	0.9176	0.9406	0.9378	0.9500
90	PSNR	21.8542	24.3237	24.4957	24.3218	24.5829
	SSIM	0.7037	0.9325	0.9484	0.9480	0.9516

**Table 2:** SSIM and PSNR of the reconstruction results of physical phantom.

Number of Views	Index	SART	SART-TV	SART-FDTV	POCS-ASD	Our Algorithm
30	PSNR	24.6119	26.2713	26.3380	26.3086	26.4178
	SSIM	0.8137	0.9109	0.9172	0.9156	0.9190
60	PSNR	25.0867	26.3724	26.4807	26.3862	26.5412
	SSIM	0.8256	0.9121	0.9204	0.9161	0.9215
90	PSNR	25.1214	26.3754	26.5431	26.4023	26.5750
	SSIM	0.8429	0.9126	0.9208	0.9185	0.9221

## Data Availability

Data are contained within the article.
